# Transportation Infrastructure, Population Mobility, and Public Health

**DOI:** 10.3390/ijerph20010751

**Published:** 2022-12-31

**Authors:** Fen Zhang, Tianyi Song, Xiang Cheng, Tianhao Li, Ziming Yang

**Affiliations:** 1 School of Economics and Management, Wuhan University, Wuhan 430072, China; 2 College of Liberal Arts and Social Sciences, City University of Hong Kong, Hong Kong 999077, China

**Keywords:** government expenditure, transportation infrastructure, population mobility, public health

## Abstract

This paper constructs an overlapping generations model, including health human capital, to investigate the impact of transportation investment on public health with population mobility. The theoretical analysis shows that there is an inverted U-shaped relationship between transportation infrastructure and population flow, which also exists between transportation and health. Health is affected by transportation from three aspects: positive output effect, negative substitution effect on public health investment, and an indirect effect through population flow. In the empirical part, considered with the infectious diseases, we found that the more intensive the traffic facilities, the greater the population flow, and therefore, the traffic facilities will have a negative impact on health. When population mortality is used to measure the level of public health, transportation improvement will significantly enhance public health with an inverted U-shaped relationship, which is consistent with the theoretical portion.

## 1. Introduction

Investment in transportation infrastructure with typical externalities has always been an important responsibility of the government, as well as an important means for the government to regulate the economy and promote economic growth [1,2]. Transportation infrastructure has a spatial spillover effect on regional economic growth through its network attributes, and a significant regional co-urbanization effect brought by transportation integration [3,4]. Nonetheless, in recent years people have started to grow concerned regarding the negative externalities of transportation, such as traffic congestion, environmental pollution, and traffic accidents. The improvement of transport infrastructure and the road network promoted the free flow and migration, which were prone to “urban diseases”, such as urban traffic congestion and air pollution [5,6]. With China growing its power in transport, the scale of inter-provincial migration had become larger and more concentrated, leading to major changes in the distribution of population by region [7]. In the background of this huge population mobility, the global outbreak of the COVID-19 pandemic in 2020 prompted people to reconsider how to develop transportation infrastructure and how to promote the process of urbanization in China [8,9].

In fact, the impact of the development of transportation infrastructure on human health is double-edged. On the one hand, the more developed the transportation infrastructure is, the more convenient the transportation network is to help people seek the external medical resources, and thus the higher the accessibility of healthcare services is. What is more, the increase of income brought by the improvement of transport could make people consume increased healthier foods and medicines [10]. Some theoretical models also incorporated infrastructure as an input factor into the health production function [11,12], and they found that infrastructure could directly affect public health. A better transportation network also helped people access health-related services provided by health care facilities, and this effect was more prominent in remote rural areas. Communication and transportation networks contributed to the popularization of health care and significantly reduced infant and maternal mortality [13,14].

Conversely, the increase in population mobility caused the population to gather in regions and cities with high-quality medical and health resources, characterized by the Grade-A Tertiary Hospitals, which not only brought social problems such as “difficult medical treatment”, but also hampered the prevention and control of infectious diseases. Higher transportation network connectivity recorded lower average height and higher death rates [15]. Scrimshaw also found that in the United States during the pre-war period, the transportation network formed by public infrastructure would connect relatively safe rural areas with disease-endemic urban areas, and thus facilitated the spreading of the diseases to the former [16]. He et al. found that highway connections drove relatively poor counties into utilizing more polluting technologies [17]. Zimran also found that there was a negative correlation between inter-regional transportation links and the citizen’s health measured by average height; the development of transportation facilities had a stronger effect on growth of population density in areas, which was suitable for wheat and corn production, and the negative impact of the transportation facilities on average height was stronger in these areas as well [18]. Therefore, transportation infrastructure could significantly affect health by affecting population mobility and local air pollution levels. However, there are few literatures to show how transportation infrastructure may negatively affect people’s health.

Hence, in the perspective of public health, it is necessary to analyze whether the improvement of transportation infrastructure has promoted or inhibited the improvement of health. Furthermore, what role did the demographics factor play in this process? Our paper attempts to combine theoretical models and empirical analysis together in explaining the impact of the development of transportation infrastructure on public health in the context of population mobility, while providing a new perspective to answer the above questions.

The remainder of this paper is as follows: Section 2 is the basic framework of the theoretical model we constructed; Section 3 is the major results of our theoretical analysis; Section 4 is the empirical analysis; and finally, Section 5 are the conclusions.

## 2. Model Setup

Dual economic theory of Lewis was widely used in research on transfer of rural labors in China. Nevertheless, most of the theoretical models ignored the impact of public health from transportation infrastructure during the process of population movement. The existing endogenous growth model with infrastructure only considered a single production sector and did not consider demographic factors [11]. Thus, it could not reflect the impact of infrastructure on the flow of population among different production sectors.

Referring to the endogenous growth model of infrastructure by Gupta and Barman, 2010 [12], our paper constructs a two-sector overlapping generations model in discussing the impact of infrastructure and population mobility on public health. We first assume that there are two distinct types of regions, namely *rural* and *urban*. The rural area consists of the primary production sector, which uses primitive labor as an input element and is free of impact of health human capital. The urban area consists of the modern production sector, which uses physical capital, health human capital, and transportation infrastructure as input elements for production.

### 2.1. Individual Decision

Consider each generation lives for two periods, child and adult. In the child period, the individual does not have an independent consumption ability nor do they make decisions on consumption, but rather they only accept the savings from the previous generation. In the adult period, an individual provides labor to the production sector and they acquire income, which will be used for consumption and savings. We do not consider the population growth; therefore, we unitized the population born in each period to be 1, as well as the adult labor population as 1. The representative individual *i* born in period *t* will be grown to an adult in period *t* + 1 and take consumption and savings, assuming the utility function is in the linear form as follows:

(1) $$u_{t}^{i}=\left(1-\gamma \right)lnc_{t+1}^{i}+\gamma lns_{t+1}^{i}$$

where $c_{t+1}^{i},\;s_{t+1}^{i}$ are adult consumption and savings, respectively, (1−γ) and γ are adult preference for consumption and savings in period *t* + 1, γ∈(0,1); as γ increases, the individual inclines to save for the next generation. The individual budget constraint is:

(2) $$c_{t+1}^{i}+s_{t+1}^{i}\leq I_{t+1}^{i}$$

$I_{t+1}^{i}$ is the total income in period *t* + 1. In accordance with solving for the optimization problem, the best solution for consumption and savings are:

(3) $$c_{t+1}^{i}=\left(1-\gamma \right)I_{t+1}^{i}$$

(4) $$s_{t+1}^{i}=\gamma I_{t+1}^{i}$$

### 2.2. Production

Different from the endogenous growth model in Gupta and Barman, 2010 [12], we considered an economy with two sectors: a rural sector and an urban sector, where the product in a rural sector was $y_{t}^{R}$, and the product in an urban sector was $y_{t}^{U}$. Assuming there is only one kind of good produced in the whole economy, then the total product $y_{t}$ in the economy is:

(5) $$y_{t}=y_{t}^{R}+y_{t}^{U}$$

The input for the first production sector is original labor $\theta_{t}$, and the efficiency of agricultural labor is A, then:

(6) $$y_{t}^{R}=F\left(\theta_{t}\right)=A\theta_{t}^{\sigma }$$

σ is the product elasticity of labor, 0<σ<1, the marginal product of input factor is greater than or equal to 0. In a perfectly competitive market, the wage of labor in the first sector $w_{t}^{R}$ is determined by the marginal product:

(7) $$w_{t}^{R}=\sigma A\theta_{t}^{\sigma -1}$$

In the urban sector, the input factor not only incudes traditional physical capital $K_{t}$, but also includes health human capital $h_{t}$ and labor $L_{t}$, which constructs efficient labor $h_{t}^{\varepsilon }L_{t}$, and also includes $G_{t}$, which is considered in the form of transportation infrastructure. Based on Agenor, 2008 and Gupta and Barman, 2010 [11,12], the production function we set is in the Cobb-Douglas form:

(8) $$y_{t}^{U}=F\left(K_{t},L_{t},G_{t}\right)=G_{t}^{\alpha }{\left(h_{t}^{\varepsilon }L_{t}\right)}^{\beta }K_{t}^{1-\alpha -\beta }$$

where α,  β are the product elasticity for the transportation infrastructure $\;G_{t}$ and the efficient labor $h_{t}^{\varepsilon }L_{t}$; 0 < α, β<1, and 0 < α+β<1.ε is an effect coefficient of health factors to human capital. The physical capital is fully discount in every period, and is formed with savings in the last period. Within each sector, the firms are perfectly competitive, the tax is $\tau y_{t}^{U}$, the tax rate τ∈(0, 1], and the rent price of other input factors are determined by their marginal productivity. Hence, the aim function of profit maximization for the second sector is:

(9) $$\pi_{t}^{U}=\left(1-\tau \right)y_{t}^{U}-R_{t}K_{t}-w_{t}^{U}h_{t}^{\varepsilon }L_{t}$$

where $w_{t}^{U}$ is the unit wage for efficient labor and $R_{t}$ is the price of the physical capital. Therefore, the rent price for each factor is:

(10) $$R_{t}=\left(1-\alpha -\beta \right)\left(1-\tau \right)G_{t}^{\alpha }{\left(h_{t}^{\varepsilon }L_{t}\right)}^{\beta -1}K_{t}^{-\alpha -\beta }$$

(11) $$w_{t}^{U}=\beta \left(1-\tau \right)G_{t}^{\alpha }{\left(h_{t}^{\varepsilon }L_{t}\right)}^{\beta -1}K_{t}^{1-\alpha -\beta }$$

### 2.3. Public Health

As the health capital is not affected by product and wage in the first sector, we assumed that expenditure for public health was solely for the labor population in the second sector. Hence, the health of representative labor in *t* period was determined by public health expenditure in the last period $H_{t-1}$ and the numbers of labor in the second sector $L_{t}$:

(12) $$h_{t}=h\left(H_{t-1},L_{t}\right)=H_{t-1}/L_{t}$$

The previous studies always considered the health capital, which was formed with public health expenditure and private investment for health, and also considered the negative effect from pollution [19]. However, the aim for our paper was the study of traffic public infrastructure on health through population flow, hence we assumed that the environment level and private investment of health were constant. Since we have assumed that the total population kept unchanged to be 1, it was that $\theta_{t}+L_{t}=1$.

### 2.4. Government

The government levies the tax on the product of the second sector,$\;\tau y_{t}^{U}$, which was distributed on transportation infrastructure $g_{t}$ and public health expenditure $H_{t}$. We assumed the service provided by transportation infrastructure was durable goods, and also had depreciation, with the depreciation rate as δ, 0≤δ≤1. The public health expenditure was exhausted one-time. Hence, the government balanced budget constraints were as follows:

(13) $$\tau y_{t}^{U}=g_{t}+H_{t}$$

(14) $$g_{t}=v\tau y_{t}^{U};H_{t}=\left(1-v\right)\tau y_{t}^{U}$$

(15) $$G_{t+1}=\left(1-\delta \right)G_{t}+g_{t}$$

The government would invest proportion *v* of its public expenditure on transportation infrastructure $g_{t}$, proportion (1 − *v*) on public health expenditure $H_{t}$. In period *t* the transportation infrastructure $G_{t}$ would be discounted in the end of period *t*, and simultaneously the government would increase new investments on transportation infrastructure $g_{t}$, which finally formed the stock of transportation infrastructure in the next period $G_{t+1}$.

Assume the labor could free flow between the two production sectors, finally the labor market would reach to equilibrium which is:

(16) $$w_{t}^{R}=w_{t}^{U}h_{t}^{\varepsilon }$$

Assume the capital completely depreciated in the current period, then the stock of physical capital in every period would be determined by the savings in the previous period under the condition of the market clearing of capital, thus we could obtain:

(17) $$S_{t}=\Sigma s_{t}^{i}=\gamma I_{t}=K_{t+1}$$

## 3. Theoretical Analysis

### 3.1. The Cross-Sector Population Flow

Considering the market equilibrium in the labor market and the wage equation in two sectors, we can obtain:

(18) $$\sigma A{\left(1-L_{t}\right)}^{\sigma -1}=\beta \left(1-\tau \right)G_{t}^{\alpha }h_{t}^{\varepsilon \beta }L_{t}{}^{\beta -1}K_{t}^{1-\alpha -\beta }$$

When the government determined to increase the proportion of investment in transportation infrastructure *v f* from $v_{0}$ in *t* period to $v_{1}$ in period *t +* 1, the market equilibrium of labor market in period *t* and *t* + 1 can be written as follows:

(19) $$\begin{array}{cc} \sigma A{\left(1-L_{t}\right)}^{\sigma -1} & =\beta \left(1-\tau \right)G_{t}^{\alpha }{\left(\left(1-v_{0}\right)\tau y_{t-1}^{u}/L_{t}\right)}^{\varepsilon \beta }L_{t}{}^{\beta -1}K_{t}^{1-\alpha -\beta }\sigma A{\left(1-L_{t+1}\right)}^{\sigma -1}\\ & ={\beta \left(-\tau \right)((1-\delta )G_{t}+v_{1}\tau y_{t}^{u})}^{\alpha }{\left((-v_{1})\tau y_{t}^{u}/L_{t+1}\right)}^{\varepsilon \beta }{L_{t+1}}^{\beta -1}K_{t+1}^{1-\alpha -\beta } \end{array}$$

In period *t* + 1, physical capital $K_{t+1}$ was determined by the product in period *t*. We assumed that period *t* remained in short-term inter-temporal equilibrium, then we had $K_{t+1}=\gamma y_{t}=\gamma y_{t-1}=K_{t}$. Similarly, in equilibrium, $y_{t-1}^{u}=y_{t}^{u},\;\delta G_{t}=g_{t-1}=v_{0}\tau y_{t}^{u}$. Hence, the Equation (19) can be sorted as:

(20) $$\begin{array}{cc} {\left(1-L_{t+1}\right)}^{\sigma -1}L_{t+1}{}^{1-\left(1-\varepsilon \right)\beta } & ={\left(1-\delta +\delta v_{1}/v_{0}\right)}^{\alpha }{\left(\left(1-v_{1}\right)/\left(1-v_{0}\right)\right)}^{\varepsilon \beta }{\left(1-L_{t}\right)}^{\sigma -1}L_{t}{}^{1-\left(1-\varepsilon \right)\beta }\\ & =P\left(v_{1}\right){\left(1-L_{t}\right)}^{\sigma -1}L_{t}{}^{1-\left(1-\varepsilon \right)\beta } \end{array}$$

where $P\left(v_{1}\right)={\left(1-\delta +\delta v_{1}/v_{0}\right)}^{\alpha }{\left(\left(1-v_{1}\right)/\left(1-v_{0}\right)\right)}^{\varepsilon \beta }$. Since σ<1 and β<1; therefore, ${\left(1-L_{t+1}\right)}^{\sigma -1}L_{t+1}{}^{1-\left(1-\varepsilon \right)\beta }$ is monotone increasing the function in $L_{t+1}$, hence the relative size between $L_{t+1}$ and $L_{t}$ is determined by the relative size between $P\left(v_{1}\right)$ and 1, when $P\left(v_{1}\right)>1$, $L_{t+1}>L_{t}$, that is, the population flows from the first sector to the second production sector in period *t* + 1; when $P\left(v_{1}\right)<1$,$\;L_{t+1}<L_{t}$, that is, the population flows from the second sector to the first sector in period *t* + 1. Then, take the derivative of $P\left(v_{1}\right)$ and set it to 0, we can obtain:

(21) $$v_{1}^{*}=\left[\alpha \delta -\varepsilon \beta \left(1-\delta \right)v_{0}\right]/\left[\alpha \delta +\varepsilon \beta \delta \right]$$

**Figure 1:**
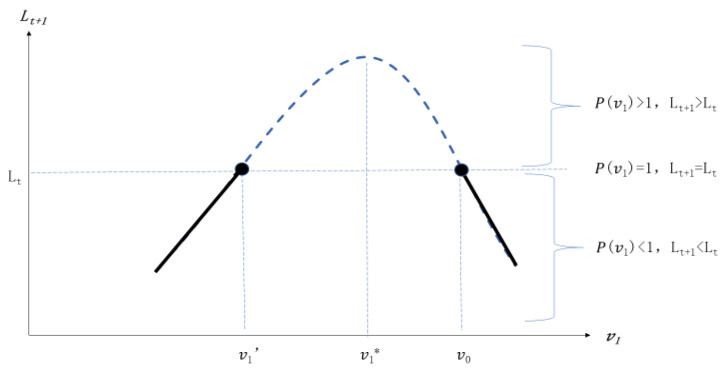
The initial investment of infrastructure is higher. Note: *v* is the proportion of public expenditure on transportation and *L* is the number of labors in second sector.

**Figure 2:**
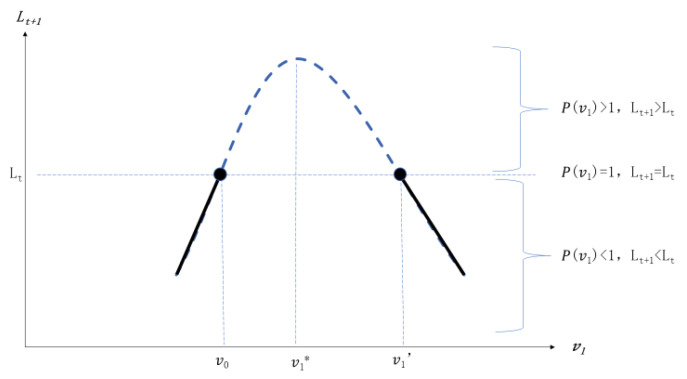
The initial investment of infrastructure is lower. Note: *v* is the proportion of public expenditure on transportation and *L* is the number of labors in second sector.

If $v_{0}<\alpha \delta /\left(1-\delta \right)\varepsilon \beta$, then $v_{1}^{*}>0$, when $0<v_{1}<v_{1}^{*}$, $P\left(v_{1}\right)$ is increasing; When $v_{1}^{*}<v_{1}<1$, $P\left(v_{1}\right)$ is decreasing it means that when the proportion of transportation infrastructure expenditure reaches to $v_{1}^{*}$, $P\left(v_{1}\right)$ reaches to its maximum, the ratio of labor in the second sector between period *t* + 1 and *t* is the highest, we call $v_{1}^{*}$ as the proportion of transportation infrastructure in the peak value of population flow ($v_{1}^{*}\leq 0$ does not have any practical meaning, we do not discuss it in this paper). When $v_{1}=v_{0}$, $P\left(v_{1}\right)=P\left(v_{0}\right)=1$, we can determine the relative size of $P\left(v_{1}\right)$ and 1 by the comparison of the relative size between $v_{1}^{*}$ and $v_{0}$. Since $P\left(v_{1}\right)$ is increasing first and then decreasing, $v_{0}\neq v_{1}^{*}$, then there exists $v_{1}^{\prime }\neq v_{0}$, which satisfies $P\left(v_{1}^{\prime }\right)=1$. Hence, we have conclusions as follows:

(1) When $\alpha \delta /\left(\alpha \delta +\varepsilon \beta \right)<v_{0}<\alpha \delta /\left(1-\delta \right)\varepsilon \beta$, $v_{0}>v_{1}^{*}>0$. Then, the initial investment of transportation infrastructure is higher (shown in Figure 1), the proportion of transportation infrastructure in period *t* + 1$\;v_{1}$ has two threshold values, and the upper threshold value is the proportion of transportation infrastructure in *t* period $v_{0}$. Hence, when $v_{1}^{\prime }<v_{1}<v_{0}$, $P\left(v_{1}\right)>1$, then $L_{t+1}>L_{t}$. That is, when the proportion of transportation infrastructure in period *t* + 1 lies between the upper threshold values and lower threshold values, compared with *t* period, some labor will flow from the first sector to the second sector and the size of labor in the second sector in *t* + 1 period is larger than the *t* period. When $v_{1}=v_{1}^{\prime }$ or $v_{1}=v_{0}$, $P\left(v_{1}\right)=1$, then $L_{t+1}=L_{t}$. That is, when the proportion of transportation infrastructure just equals to the upper threshold value or lower threshold value, then the labor size in both sectors maintains unchanged. When $v_{1}<v_{1}^{\prime }$ or $v_{1}>v_{0}$, $P\left(v_{1}\right)<1$, then $L_{t+1}<L_{t}$. That is, when the proportion of transportation infrastructure in period *t* = 1 does not lie between the upper threshold value and lower value, compared with period *t*, some labors will flow from the second sector to the first sector, and the size of labor in period *t* + 1 will be lower than in period *t*.

(2) When, $v_{0}<\alpha \delta /\left(\alpha \delta +\varepsilon \beta \right)$, $v_{1}^{*}>v_{0}>0$, the initial investment of public infrastructure stays in the low level, then $v_{1}$ still has two threshold values and the lower threshold values is $v_{0}\;$(shown in Figure 2). Hence, when $v_{0}<v_{1}<v_{1}^{\prime }$, $P\left(v_{1}\right)>1$, then $L_{t+1}>L_{t}$, where some part of labor flow from the rural sector to the urban sector and the labor size increases in period *t* + 1. When $v_{1}=v_{1}^{\prime }$ or $v_{1}=v_{0}$, $P\left(v_{1}\right)=1$, then $L_{t+1}=L_{t}$, the labor does not exit the cross-sector flow. When $v_{1}<v_{0}$ or $v_{1}>v_{1}^{\prime }$, $P\left(v_{1}\right)<1$, then $L_{t+1}<L_{t}$, compared with period *t*, some labor will flow from the second production sector to the first production sector, and the labor size in the second sector will be decreasing.

**Proposition** **1.** *In the population flow among different sectors exists a threshold, which is determined by the proportion of investment in the transportation infrastructure. When the investment proportion lies within the proper range, it will improve the labor flow from the rural sector to the urban sector. When the proportion of investment is higher or lower, it will lead to a backflow of labor from the urban sector to the rural sector.*

### 3.2. Transportation Infrastructure, Population Flow, and Public Health

Comprehensively considering the Equations (12) and (14), we can express that $h_{t+1}=\left(1-v_{1}\right)\tau y_{t}^{u}/L_{t+1}$, and further, $h_{t+1}/h_{t}=[(1-v_{1})/\left(1-v_{0}\right)]\left(L_{t}/L_{t+1}\right)$. Hence, as the adjustment of proportion of transportation infrastructure and the cross-sector population flow, the health status of representative labor will change. When $v_{1}>v_{0}$ and $L_{t+1}>L_{t}$, $h_{t+1}<h_{t}$, that is, if the investment and density of the transportation infrastructure increase the population flow to modern production sector, then the health damages for the crowding effect from the dense population (correspond to situation (2): when $v_{0}<v_{1}<v_{1}^{\prime }$, $h_{t+1}<h_{t}$). In reverse, if the government reduces the investment in transportation infrastructure, the population will flow backward, which is beneficial for the health improvement (corresponding to situation (2): when $v_{1}<v_{0},$ $h_{t+1}>h_{t}$; and situation (1): when $v_{1}<v_{1}^{\prime }$ < $v_{0}$, $h_{t+1}>h_{t}$). For other situations, the investment allows for the health change to be more complicated, which cannot be explained by the short-term equilibrium from the equations, hence, we would discuss the effect of public infrastructure and population flow on health in the perspective of long-term equilibrium.

#### 3.2.1. The Long-Term Driving Factors of Transportation Infrastructure on Flow

With the long-term equilibrium condition in the capital market, we can obtain the equation between the proportion of public expenditure in transportation infrastructure (*v*) and the labor in the second production sector:

(22) $$\delta^{\alpha }\sigma^{1-\alpha -\varepsilon \beta }\gamma^{\alpha +\beta -1}A^{\left(1-\varepsilon \right)\beta }{\left(1-L\right)}^{\left(\sigma -1\right)\left(1-\varepsilon \right)\beta }L^{1-\alpha -\beta }{\left[1+\left(\frac{\sigma }{\beta \left(1-\tau \right)}-1\right)L\right]}^{\alpha +\beta -1}\;={\left[\beta \left(1-\tau \right)\right]}^{1-\alpha -\varepsilon \beta }\tau^{\alpha +\varepsilon \beta }v^{\alpha }{\left(1-v\right)}^{\varepsilon \beta }$$

The right-hand side of Equation (22) is a function of v; if we let its first derivative be equal to 0, we can obtain $v^{*}=\alpha /\left(\alpha +\varepsilon \beta \right)$, when $v<v^{*}$; the RHS of the equation is strictly increasing in v; when $v>v^{*}$, the RHS of equation is decreasing in v. Because of 0<σ<1 and 0<α+β<1, the LHS is strictly increasing in L. Hence, when $v<v^{*}$.

**Figure 3:**
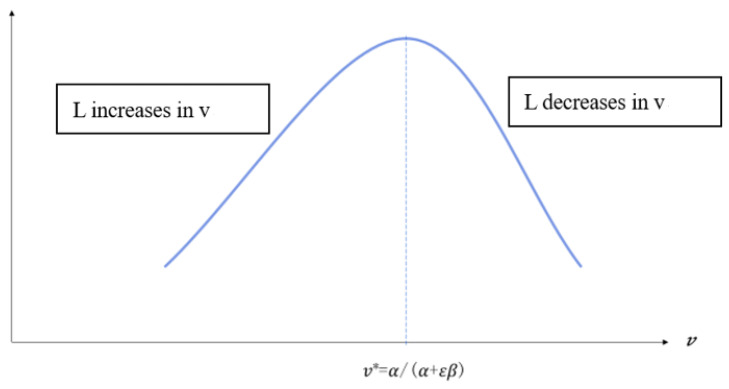
The transportation infrastructure and population flow. Note: *v* is the proportion of public expenditure on transportation and *L* is the number of labors in second sector.

*L* is increasing in v, then (1 − *L*) will decrease, which means the size of labor in the second production sector increases, and the first sector decreases, and the product in the second sector also increases with the decrease of the product in first sector. When σ>β(1−τ), the total product increases; when $v>v^{*}$, *L* decreases with v, that is, the size of labor in the second sector decreases and the size of labor in the first sector increases, the product of first sector increases as the decrease of the second sector occurs; when σ>β(1−τ), the total product decreases. Hence, as Figure 3 shows the size of labor *L* has an inverse U-shaped relationship with the proportion of expenditure in transportation infrastructure *v*.

When $v=v^{*}=\alpha /\left(\alpha +\varepsilon \beta \right)$, the size of labor in the sector production sector reaches to its maximum. Then, the proportion of investment on the transportation infrastructure equals to the ratio between elasticity of investment on transportation infrastructure to product (α) and elasticity of government expenditure (including investment on transportation infrastructure and public health) in the second sector to product (α+εβ). This is because the labor flow between the two sectors is driven by wage discrepancy as the input factors of the second production sector, transportation infrastructure investment, and public health expenditure affecting the wage. This occurs when the proportion of transportation infrastructure and public health expenditure equal to the elasticity of these two inputs to production and the size of labor reaches to maximum value. Hence, we obtain the conclusion as follows:

**Proposition** **2.** *In the long-run, if the proportion of investment in transportation infrastructure is lower than its elasticity of the product, then the proportion of transportation infrastructure increases and the labor would flow from the first production sector to second production sector, and vice versa.*

#### 3.2.2. The Effect of Infrastructure on Health through Population Flow

First, we analyze the relationship among transportation infrastructure, population flow, and health and we derive health production function with respect to *v*:

(23) $$\partial h/\partial v=h_{H}*\partial H/\partial v+h_{L}*\partial L/\partial v$$

Then substituting (23) into Equation (14), we can further obtain:

(24) $$\partial h/\partial v=h_{H}\left(1-v\right)\tau \partial y^{U}/\partial v-h_{H}\tau y^{U}+h_{L}*\partial L/\partial v$$

Hence, the effect of investment in transportation infrastructure on health can be divided into three parts: the first term in RHS of Equation (24) measures its direct effect, which comes from the tax income with the change of product, with this effect being positive; the second term is a direct substitutive effect between traffic investment and health investment, with its effect being negative; the last term is also an indirect population effect, which transit from the population flow, because the relationship between *L* and *v* acquires an inverse-U shape, thus the effect from this part is uncertain, and finally affects the total effect of traffic on health.

Further, combined with the equilibrium in the labor market, we can obtain the long-term equilibrium, which is:

(25) $$h=\frac{\sigma A\tau }{\beta \left(1-\tau \right)}{\left(1-L\right)}^{\sigma -1}\left(1-v\right)$$

Take the derivative of *h* on *v* with the Equation (25):

(26) $$\frac{\partial h}{\partial v}=\frac{\sigma A\tau }{\beta \left(1-\tau \right)}{\left(1-L\right)}^{\sigma -1}\left[\left(1-\sigma \right)\frac{1-v}{1-L}*\frac{\partial L}{\partial v}-1\right]$$

**Figure 4:**
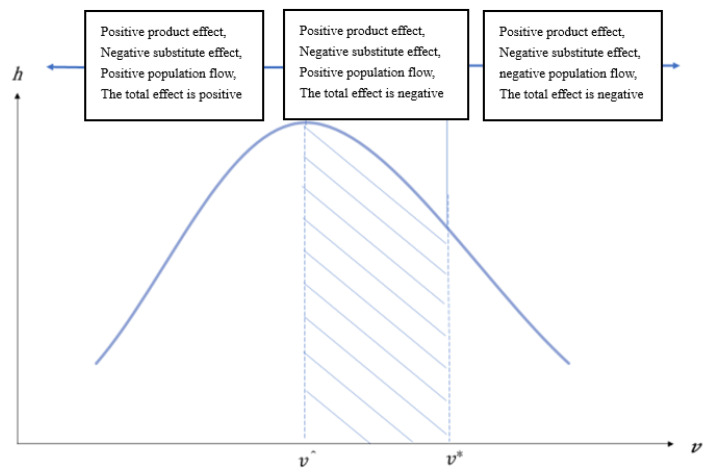
Transportation infrastructure, population flow, and health. Note: *v* is the proportion of public expenditure on transportation and *h* is health capital.

When (1−σ)(1−v)/(1−L)∗∂L/∂v>1, ∂h/∂v>0, the health human capital *h* is increasing in *v*; when (1−σ)(1−v)/(1−L)∗∂L/∂v<1, ∂h/∂v<0, the health *h* will decrease with *v*. In Equation (22), we know that *L* and *v* is an inverse U-shaped. Although we cannot write out the explicit form of function of *L* on *v*, since 0<σ<1, when ∂L/∂v≤0, that is, when $v\geq v^{*}=\alpha /\left(\alpha +\varepsilon \beta \right)$, ∂h/∂v<0. In other words, when *v* = $v^{*}$, health *h* has a negative relationship with *v*. Hence, we could find that health *h* also has an inverse U-shaped relationship with *v*, and its turn point $\hat{v}$ is less than the turning point of population flow $v^{*}$, that is $\hat{v}<v^{*}$. When *v* is less than $\hat{v}$, the convenience of traffic is beneficial to the population flow. Then, the positive effect will dominate in the outcome of transportation infrastructure on health. If *v* is larger than $\hat{v}$, then the negative effect will be dominant (shown in Figure 4).

**Proposition** **3.** *The proportion of investment on infrastructure has an inverse U-shaped with population flow and health. That is, as the increase of investment on transportation infrastructure occurs, the population flows to the modern production sector and if the positive effect of population flow on health is dominated, then the transportation infrastructure has a positive effect on health. If the investment on infrastructure occurs in the middle level, although the population still flows to modern sector because of the weak positive effect, the effect of transportation infrastructure on health will be negative. That is, the health deteriorates with the increase of population. If there is an over-investment on transportation infrastructure, then the population will backward flow to the traditional sector, and the negative effect will be dominant, with the transportation infrastructure having a negative effect on health as a whole.*

## 4. Empirical Analysis

### 4.1. Model Specification and Variables

We further examined the impact of infrastructure and population mobility on health through empirical analysis from two perspectives. Firstly, in the context of general infectious diseases, we analyzed the impact of transportation infrastructure and population mobility on the spread of diseases. Secondly, we discussed the impact of infrastructure and population flow on public health in the case of the COVID-19. The description of used variables is shown in Table 1. The empirical model employed in the paper was based on the health production function proposed by Grossman, 1972 [20]. Therefore, the basic model for empirical analysis is as follows:

$$health_{it}=\alpha_{0}+\alpha_{1}pubinf_{i,t}+\alpha_{2}pop_{i,t}+\sum \beta X_{it}+\varepsilon_{it}$$

### 4.2. The Case of Infectious Diseases

The impact of transportation infrastructure and population movement on the spread of ordinary infectious diseases would be discussed first. Considering the fact that there may exist an endogenous relationship between GDPs per capita, health expenditure per capita, and the public health of a region, we analyzed with lagged term of these variables and adopted a logarithm for them. Considering that the provincial panel data included both the time dimension and the cross-sectional dimension, there may be heteroscedasticity, serial correlation, and cross-section correlation, and the Hausman test rejected random effects, while a fixed effects model was adopted for regression analysis.

Table 2 summarizes the regression results of the impact of transportation infrastructure and population movement on the incidence of infectious diseases. In columns (1) to (3), railway density was used in quantifying transportation infrastructure. When the impact of railway network accessibility on population mobility was ignored, the regression result (1) exhibited consistency with the literature. Areas with a higher population density had a significantly higher incidence of infectious diseases, yet the population mobility reduced the incidence. The regression result (2) considered the impact of railway density on population mobility. The results showed that after introducing the interaction terms between railway density and floating population, the impact of population density on public health was no longer significant, while railway density and floating population had an impact on the incidence of infectious diseases. Their impact coefficients were both significant and positive at 1%, indicating that a high-density railway infrastructure and a high level of population mobility would both increase the incidence of infectious diseases. Meanwhile, the interaction term between railway density and floating population was significant while negative, implying that the more developed the railway network, the higher the population mobility of a region, thereby lowering the incidence of infectious diseases of the same region. This may be explained as follows: a convenient system of transportation infrastructure could assist people in seeking a better external medical and disease prevention and control environment when the disease spreads, thereby reducing the risk of infection. At the same time, the turn point of a logarithm railway could be calculated from the coefficients of the individual terms and the square term to 18.62. Thus, the turn point of the railway density was about 122,057,157, which was much higher than the existing railway density level in all of the regions, while they were all located on the left side of the turn point.

We also examined the impact of road accessibility on public health. The regression result (4) showed that compared with railways, there was no significant correlation between road density and public health, and a convenient road network could not lead to a higher level of population mobility in lowering the incidence of infectious diseases in a densely populated areas as railways did. Based on our analysis, it could be concluded that the population density factor was indeed not conducive to the prevention and control of infectious diseases, while the transportation infrastructure (characterized by railways) would serve as the transmission vector of infectious diseases, to some extent. Nonetheless, our analysis also showed that the factor of population mobility, which was introduced by the convenience of transportation, did not necessarily increase the local risk of infectious diseases.

We also used population mortality in quantifying the level of public health and as an explained variable to analyze the relationship between transportation infrastructure, population mobility, and health in a more general context. The regression result (5) showed that the coefficient of influence of railway density on mortality was significant and negative at 5%, while the coefficient of the square term of railway density on mortality was significant and positive at 1%. This indicated that in general, the convenience of transportation infrastructure had a significant positive impact on public health. The higher the density of transportation infrastructure, the lower the mortality rate and the higher the public health level would be. Notwithstanding, the marginal effectiveness of this positive impact was diminishing and appeared as an inverted U-shape to some extent. Nationwide, the impact of the mobile population on the mortality rate was overall not significant. Our comparison found that the density of transportation infrastructure had a significant positive impact on the incidence of infectious diseases, and a significant negative impact on the mortality rate. This demonstrated that a convenient system of transportation infrastructure could improve public health in ordinary situations, yet an adverse effect in the case of infectious diseases. Meanwhile, the mobile population had a significant impact on the incidence of infectious diseases but not on the mortality rate, which implied that the mobile population played a vital role in the spread of infectious diseases.

According to the density of transportation infrastructure and the degree of population mobility, we grouped the samples to perform a heterogeneity analysis. The results are shown in Table 3. Group 1 was further divided into three equal subgroups of low, medium, and high, in accordance with the density of infrastructure in the regions. The results are listed in columns (1)–(3). We found that the density of transportation infrastructure and the floating population both had a positive and significant impact on the incidence of infectious diseases, except for areas with the medium infrastructure density. Group 2 was divided into three equal subgroups: net population outflow, relative equilibrium, and net population inflow. As shown in columns (4)–(6), the results demonstrated that, compared to areas with relatively stable populations, in the areas with greater population mobility, the inflow and outflow of the population both significantly increased the incidence of infectious diseases, and negatively affected the level of public health. Albeit in general, the population could flow with the help of a convenient system of transportation infrastructure, thereby alleviating the incidence of infectious diseases.

### 4.3. Further Discussion

**Figure 5:**
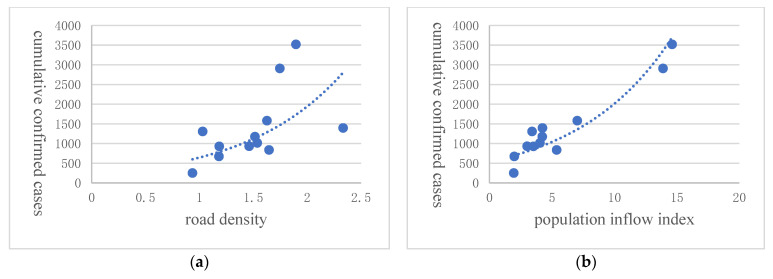
Different cities in Huber Province. Note: Road density (**a**) comes from China’s National Bureau of Statistics. The number of confirmed cases is from China’s National Health Commis-sion, and the population inflow index (**b**) is from Baidu Map. These data are in city level of Hu-bei. The dashed line is the quadratic fitted line.

Considering the fact that the Novel Coronavirus Epidemic is more distinctive than general infectious diseases, we further discuss the impact of transportation infrastructure and population mobility on public health. In the context of the COVID-19 outbreak, we conducted a correlation analysis among the density of transportation infrastructure, population movement, and the number of the confirmed cases of COVID-19. This analysis was carried out on two aspects, intra Hubei province and inter-province level, respectively. In Hubei Province, we used the migrant population scale index of Wuhan from 10 January to 23 January 2020 multiplied by the corresponding percentage of the migrant destinations (as cities) in quantifying the population flow from Wuhan to other cities in Hubei. These numbers were retrieved from the Big Data Platform on Migration of Baidu Map, while the data on the number of confirmed cases of the pandemic sources from information released by the Hubei Provincial Health Commission. Due to the lack of railway mileage data of separate cities, we only used road density to quantify the level of transportation infrastructure development. The data are from China Statistical Yearbooks and statistical bulletins of the respective cities. From the trend lines shown in Figure 5, we can see that in all cities in Hubei Province (except Wuhan) the cumulative number of COVID-19 cases was positively correlated with road density, as well as migration inflow. Hence, we could conclude that the more developed the transportation infrastructure and the higher the migration inflow, the more extensive that the spread of the Coronavirus would be.

**Figure 6:**
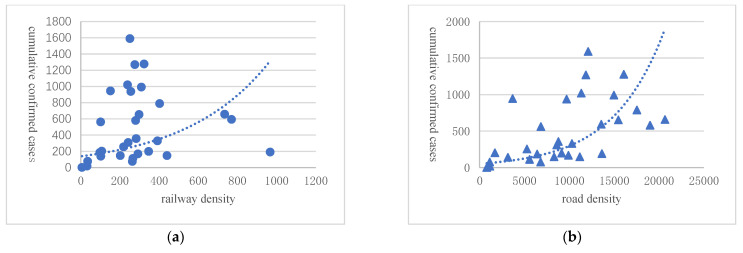
The confirmed cases in COVID 19 and transportation infrastructure in different prov-inces in China. Note: Railway density (**a**) and Road density (**b**) are from China’s National Bureau of Statistics. The number of confirmed cases is from China’s National Health Commission. The dashed line is the quadratic fitted line..

**Figure 7:**
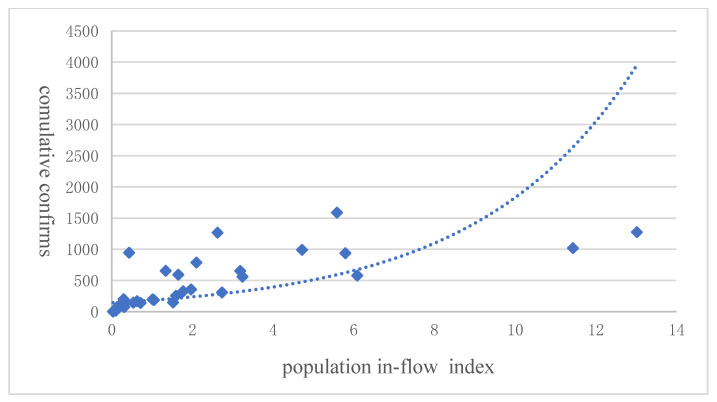
The confirmed cases in COVID 19 and the population flow from Hubei in different provinces. Note: The number of confirmed cases is from China’s National Health Commission, and the population inflow index is from Baidu Map. The dashed line is the quadratic fitted line.

At the inter-provincial level, the scale index of Hubei’s outward migration from 10 January to 23 January 2020 is multiplied by the share of respective migration destinations in quantifying the scale of outward migration of Hubei. The data on the number of confirmed cases of COVID-19 sourced from the information released by the Health Commissions of corresponding provinces, whereas the data on railway and road density are from the China Statistical Yearbook. The scatter graphs are shown as Figure 6 and Figure 7. From the trend lines, we could observe that in the provinces other than Hubei, the cumulative number of confirmed cases of the Coronavirus was positively correlated with both road density, railway density, and the inflow migration index. This was consistent with the situation we observed inside the Hubei Province. That is, the more developed the transportation infrastructure and the higher the migration inflow was, the more extensive the spread of the virus would be.

## 5. Conclusions

Based on the above analysis we found that, in the case of infectious diseases, the more convenient the transportation infrastructure and the bigger the scale of migration, there would be a higher incidence of infectious diseases. Notwithstanding, the interaction between transportation infrastructure and population movement had a positive impact on the incidence of infectious diseases. Nonetheless, in our correlational analysis in the case of the COVID-19, the cumulative number of confirmed new cases was both positively correlated with the density of transportation infrastructure and the inflow migration index. One possible explanation for this difference could be that the conventional infectious diseases fell largely within the coverage of the existing medical and disease prevention and control system, under which people could use convenient transportation infrastructure in seeking external medical help and thus reduce the risk of these diseases. Due to little knowledge and the highly contagious nature of the novel Coronavirus, the existing medical system acted relatively ineffectively in reducing the risk of this disease. Therefore, the correlation among the density of transportation infrastructure, the mobile population, and the spread of this new pneumonia were stronger than that of other general infectious diseases.

Nevertheless, under the case of non-infectious diseases with general health indicators, the more convenient the transportation is the lower the mortality rate and the higher the public health level would be, and there would be an inverted U-shaped relationship between the transportation infrastructure and the public health level. This was consistent with the predictive result we obtained in the theoretical part.

Hence, regions with a high density of transportation infrastructure and high population mobility would face a higher risk of infectious diseases, especially during the spreading stage. Therefore, the implementation of traffic control and population movement restrictions during the exponential spreading stage of diseases played a certain role in controlling the ultimate outbreak of the epidemic. Simultaneously, the outflow migration trend depicted by big data sets was shown to be positively correlated with the number of confirmed COVID-19 cases, which provided effective information for epidemic control and public health risk management. Therefore, we should use big data technologies in epidemic prevention, control, and public emergency management.

For non-infectious diseases, the density of transportation infrastructure would have an inverted U-shaped relationship with the level of public health. Though regions in China are currently in the left half of the inverted-U line, that is, in the positive impact stage considered to be the diminishing effect of transportation infrastructure on public health improvement during its continuous development, we should implement some measures in the medical system. This should be done with consideration to the regional factors, such as further promotion to the construction of medical systems, enlarging the scale of medical and health investment, striving to reduce the regional differences, and promoting the equalization of medical and health services, as well as effectively improving the standard of public medical and health services.

## Figures and Tables

**Table 1:** Description of data and variables.

Variables	Definition and Interpretations	Mean	Std.Err	Min	Max
crfb	Reported morbidity rates of class A and B Infectious (1/100,000)	264.38	101.59	91.24	738.19
death	Mortality rate (%)	6.13	0.82	4.14	11.85
railway	Length of railways/area of province	192.78	168.29	8.06	875.81
highway	Length of highways/area of province	6108.16	4495.83	192.52	20,809.02
mob	Permanent Residents-registered population	17.26	441.71	−1736.99	1958.11
popd	Permanent Residents/area of province	419.19	579.99	6.88	3825.90
soot	Volume of Industrial soot(dust) emission	32.01	23.705	1.30	145.07
green	Green areas per capita	12.01	9.54	1.59	57.57
gdp	GDP per capita	24,580.35	21,494.2	2215	108,000
pubh	Per capita Expenditure for medical and public health	284.79	311.72	15.06	1706.68

**Table 2:** Transportation infrastructure, population flow, and the incidence of infectious disease.

	Dependent Variable: Incidence of Infectious Disease				Mortality Rate
	FE(1)	FE(2)	FE(3)	FE(4)	FE(5)
lnrailway	−0.098 (0.369)	3.699 ** (0.013)	5.276 *** (0.001)		−2.856 ** (0.038)
lnrailway^2			−0.128 *** (0.000)		0.296 *** (0.000)
lnhighway				−1.512 (0.409)	
lnhighway^2				−0.026 (0.193)	
lnmob	2.830 (0.000)	2.913 *** (0.000)	2.602 *** (0.000)	1.202 (0.381)	−0.159 (0.869)
lnrailway * lnmob		−0.506 ** (0.011)	−0.555 *** (0.005)		0.011 (0.949)
lnhighway * lnmob				0.302 (0.192)	
lnpopd	2.628 *** (0.001)	−0.696 (0.644)	−1.258 (0.397)	4.274 *** (0.004)	−0.429 (0.167)
lnpopd * lnmob	−0.443 *** (0.000)		0.092 (0.647)	−0.644 *** (0.001)	
lnsoot	−0.056 (0.167)	−0.061 (0.126)	−0.078 * (0.051)	−0.010 (0.804)	0.073 (0.248)
lngreen	0.002 (0.978)	−0.013 (0.836)	0.013 (0.825)	−0.037 (0.527)	0.129 (0.151)
L_lngdp	0.379 *** (0.000)	0.384 *** (0.000)	0.330 *** (0.001)	0.127 (0.228)	−0.794 *** (0.000)
L_lnhcare	−0.167 *** (0.005)	−0.167 *** (0.005)	−0.138 ** (0.019)	−0.133 ** (0.022)	0.406 *** (0.000)
observations	406	406	406	406	522
$R^{2}$	0.092	0.108	0.142	0.156	0.147
*F* statistic	4.70 (0.000)	4.97 (0.000)	6.05 (0.000)	6.76 (0.000)	9.28 (0.000)

Note: Standard errors are in parenthesis; *** *p* < 0.01, ** *p* < 0.05, * *p* < 0.1. ln is the prefix for each variable, which means taking logarithm for each variable. The prefix “L-” means taking lags for the variable lngdp and lnhcare. lnrailway^2 is the square term of lnrailway, lnhighway^2 is the square term of lnhighway.

**Table 3:** Heterogeneity analysis.

	Dependent Variable: Incidence of Infectious Disease					
	Panel 1: The Density of Public Infrastructure			Panel 2: The Population Flow		
	High FE(1)	Middle FE(2)	Low FE(3)	Net in Flow FE(4)	Balanced FE(5)	Net Out Flow FE(6)
lnrailway	7.942 *** (0.000)	6.023 (0.114)	11.802 *** (0.000)	7.545 *** (0.000)	−12.943 (0.205)	−2.412 (0.307)
lnmob	2.758 ** (0.046)	3.913 (0.121)	5.899 *** (0.000)	6.390 *** (0.000)	−8.325 (0.224)	2.886 *** (0.001)
lnrailway^2	−0.422 *** (0.004)	0.069 (0.690)	−0.365 *** (0.000)	−0.122 ** (0.019)	−0.092 (0.207)	0.555 *** (0.002)
lnrailway * lnmob	−0.477 ** (0.047)	−0.904 * (0.061)	−1.232 *** (0.000)	−0.845 *** (0.000)	1.793 (0.179)	−0.466 *** (0.002)
Control variables	√	√	√	√	√	√
observations	140	126	140	140	126	140
$R^{2}$	0.285	0.203	0.320	0.449	0.236	0.288
*F* statistic	5.34 (0.000)	3.05 (0.003)	6.32 (0.000)	10.97 (0.000)	3.71 (0.000)	5.42 (0.000)

Note: Standard errors are in parenthesis; *** *p* < 0.01, ** *p* < 0.05, * *p* < 0.1. ln is the prefix for each variable, which means taking logarithm for each variable. lnrailway^2 is the square term of lnrailway

## Data Availability

The data that support the findings of this study are openly available in National Bureau of Statistics of China.
